# Validity and reliability of the Flare-OA scale for hip and knee osteoarthritis in a Turkish population: item reduction with Rasch analysis

**DOI:** 10.1007/s00296-025-05914-3

**Published:** 2025-07-03

**Authors:** Mehmet Tuncay Duruöz, Halise Hande Gezer, Jonathan Epstein, Marc Soudant, Francis Guillemin

**Affiliations:** 1 Marmara University School of Medicine, Rheumatology, Istanbul, Türkiye; 2 CHRU, Inserm, Université de Lorraine, CIC Epidémiologie Clinique, Nancy, France; 3 Université de Lorraine, Inserm, UMR 1319 INSPIIRE, Nancy, France

**Keywords:** Osteoarthritis, Knee, Osteoarthritis, Hip, Disease flares, Psychometrics, Surveys and questionnaires, Quality of life

## Abstract

**Objectives:**

The Flare-OA questionnaire is a self-reported instrument developed to assess flare in individuals with knee and/or hip osteoarthritis. This study aimed to translate and culturally adapt both the original and short versions of the Flare-OA into Turkish.

**Methods:**

The Turkish version of the questionnaire was obtained through a process of cross-cultural adaptation and translation. Patients aged 45 years or older with clinically and radiologically confirmed OA of the knee or hip were recruited. The Flare-OA scale, originally consisting of 33 items, was shortened to 19 items and then to a final 16-item version through Rasch analysis. The internal consistency of the Flare-OA was measured using Cronbach’s alpha, and its stability over time was tested by evaluating test–retest reliability over a 15-day interval in patients with no clinical changes. The sensitivity to change was determined by calculating the standardized response mean (SRM) in those who reported symptom variation during the follow-up. Convergent validity was assessed by analyzing the correlations between the scale and previously validated measures, including the Hip Disability and Osteoarthritis Outcome Score (HOOS-PS), the Knee Injury and Osteoarthritis Outcome Score (KOOS), and the Mini-Osteoarthritis Knee and Hip Quality of Life Questionnaire (Mini-OAKHQOL).

**Results:**

The study included 185 participants, of whom 71.9% were women, with a mean age of 63.2 years (SD:9.1). Of these, 160 patients (86.5%) had knee OA and 25 (13.5%) had hip OA. In the past four weeks, 70 patients (37.8%) reported a worsening of symptoms in the affected joint. Cronbach’s alpha coefficient was 0.987 (95% CI 0.984–0.990) for the 33-item and 0.972 (95% CI 0.966–0.978) for the 16-item. The intraclass correlation coefficient was 0.913 and 0.912 for the test-retest reliability (n=79) of the 33- and 16-item tests, respectively. Sensitivity to change was good in 9 patients with flare improvement [SRM 1.2 (95% CI 0.6–1.7), SRM 1 (95% CI 0.5–1.5), for 33- and 16-items, respectively] over the period. Discriminant validity was supported by statistically significant score differences between patients with and without flare for both the 33-item [36.2; 95% CI 29.9–42.6; SEM: 8; p<.0001] and the 16-item [36.7; 95% CI 30.3–43.0; SEM: 8.1; p<.0001] versions. There was a significant and negative correlation between the Flare-OA score and KOOS and mini-OAKHQOL (p<0.05). 16-item Rasch modeling allowed us to reduce the questionnaire to a 16-item version with good fit and a satisfactory interval scale.

**Conclusion:**

The Turkish versions of the Flare-OA questionnaires (33- and 16-item) showed high reliability, validity, and clinical utility in evaluating flares in knee and hip OA. The 16-item version appears especially useful for routine use, although further validation is needed due to the limited sample size in the hip OA subgroup.

**Supplementary Information:**

The online version contains supplementary material available at10.1007/s00296-025-05914-3.

## Introduction

Osteoarthritis (OA) is the most prevalent arthritic condition, leading to chronic pain and functional disability in adults. Although it primarily affects the hips, knees, and hands, it has the potential to involve any joint [1, 2]. Based on the Global Burden of Disease study, the worldwide prevalence of OA in 2020 was 595 million individuals, which accounts for 7.6% of the global population. Osteoarthritis cases are expected to increase by 74.9% for the knee and 78.6% for the hip by 2050 as compared to 2020. This situation is expected to continue with the aging of the global population, and the contributing factors such as obesity [3–7].

Osteoarthritis is typically characterized by symptoms such as pain in the affected joint, stiffness, swelling, crepitus, reduced mobility, structural deformities, and weakness. Osteoarthritis flares are sudden changes in symptoms and clinical findings, and these flares can be disabling. Flare-up experiences vary and may exist on a continuum. For example, some persons who have a flare-up may not have any swelling or discomfort. Similar to symptom patterns, the triggers and consequences of OA flares may differ both across individuals and within the same individual over time throughout the disease progression [8–10].

The term “osteoarthritis flare” refers to a wide range of symptoms, not simply an exacerbation of pain alone. In recent years, flares have been more thoroughly comprehended in a variety of diseases, including OA, and are frequently used as a clinical study outcome. Nevertheless, there is no standardized definition or measurement instrument for OA flares, despite the fact that a variety of assessments have been tested to evaluate them [11, 12]. A universally accepted definition of flare is essential for ensuring consistency across studies, improving inter-researcher communication, enabling accurate identification of underlying mechanisms and therapeutic targets, and guiding clinicians in the prompt recognition and management of disease flares [12]. In order to support this gap, OMERACT-OARSI developed a definition and the core set of domains addressing flare. This is set for a flare of OA, which consists of pain, swelling, stiffness, psychological aspects, and the effect of symptoms [13]. After the core set was established, this group developed a flare scale for hip and knee OA. The recently developed Flare-OA self-reported questionnaire, consisting of 33 items, is the first tool designed to assess the frequency and intensity of flares in individuals with knee and hip OA. The development of the tool involved many processes, including the use of mixed techniques and a content-driven approach [14]. The initial scale consisted of 33 items and through the use of confirmatory component analysis, the scale was reduced to 19 items. Further refinement using the Rasch model resulted in a final scale consisting of 16-items. Following this development process and final analysis, the Flare-OA-33, 19, and 16 self-report questionnaires were ready for use in clinical research.

This study aimed to provide a Turkish translation and cultural adaptation of both the 33- and 16-item Flare-OA scales and to investigate the validity and reliability of the adapted forms.

## Materials and methods

### Patients

Patients with knee or hip OA were recruited from the rheumatology clinic at Marmara University. Ethical approval for the study was obtained from the Ethics Committee of Marmara University Medical School (Approval No: 847, Date: 09.2020), and the research was conducted in accordance with the principles of the Declaration of Helsinki. All patients were informed about the study procedure and gave written consent to participate. We confirm that permission to translate and validate the Flare-OA into Turkish was obtained from the original developers of the questionnaire. The necessary copyright and usage rights were granted prior to the initiation of the study.

Patients aged 45 years or older, diagnosed with symptomatic knee or hip OA based on clinical and radiological findings in accordance with ACR criteria [15, 16] and confirmed by a doctor, regardless of the onset date of OA, were included. Patients who could not read and understand documents written in Turkish and patients with OA of both hip and knee were excluded. The sample size was determined based on literature recommendations, aiming for 5 to 7 times the number of items in the scale.

Demographic and clinical features, including age, gender, affected joints, presence of prosthesis, additional rheumatic diseases, and medications, were recorded at outpatient visits by online questionnaire, which was administered between September 2021 and July 2022.The questionnaire was self-administered electronically via tablets provided in the clinic, and data were collected in real-time during patient visits. Since the questionnaire required responses to all items before submission, there were no missing data. This study followed the 16-item EULAR–OMERACT recommendations for the design and reporting of survey-based research [17].

## Instrumentation

The main question for assessment of flare was, “Have you had a flare-up of your osteoarthritis in the last 4 weeks?“. This question was answered as yes or no. The Flare-OA is a patient-reported outcome measure designed to evaluate flare status over the preceding four weeks. It includes 33 items rated on an 11-point numeric scale (0 = Not at all agree, 10 = Definitely agree), covering five flare-related domains: pain (6 items), swelling (2 items), stiffness (2 items), consequences of symptoms (sleep, concentration, activity, need for help, walking; 14 items), and psychological effects (9 items). (Table 1) [11]. The model with 33 items had inadequate fit indices, with all components demonstrating loadings over 0.4 within their respective domains. Certain items were linked to many domains, and the “consequences of symptoms” domain had a robust correlation with the other four domains. Following content evaluations and index modifications for the confirmatory factor analysis (CFA), 14 items were eliminated, resulting in the Flare OA scale being condensed to 19 items by component analysis and content methodology (RMSEA = 0.06; CFI = 0.96; TLI = 0.94) [14]. In the next step, Rasch analysis was applied to reduce the questionnaire to 16 items with a valid structure [18]. The 16-item version of Flare OA demonstrated acceptable construct validity based on CFA, along with evidence of convergent validity through its correlations with the HOOS-PS, KOOS, and Mini-OAKHQOL [19].

**Table 1:** Domains and definitions of knee/hip OA flare [11]

Domain	Definition
Pain	A distinct change in pain that is more severe and lasts longer is particularly heightened with physical activity and persists with rest.
Swelling	An increase in size or feeling of fullness of the joint.
Stiffness	Increased or prolonged stiffness of the joint that does not resolve with movement
Psychological aspects	Alterations in mood, including depressive symptoms, greater anxiety, greater irritability, and/or low morale, are consequences of the symptoms during a flare.
Impact of symptoms	A change in the ability to perform daily activities requires new adaptations and strategies due to the increase in pain, swelling, stiffness, fatigue, and sleep disturbance related to the flare.

The KOOS (Knee Injury and Osteoarthritis Outcome Score) is a joint-specific, patient-reported questionnaire designed to assess knee symptoms,physical functioning, and health-related quality of life in individuals diagnosed with knee osteoarthritis [20]. The Turkish version of KOOS has also been shown to be valid and reliable in knee OA [21].

The Hip Disability and Osteoarthritis Outcome Score-Physical Function Short Form (HOOS-PS) is a commonly used measuring instrument for assessing physical function in individuals with hip OA. It aims to evaluate patients’ ability to perform daily activities and the limitations they experience due to hip pain. The HOOS-PS is a shortened version of the HOOS, comprising five items compared to the original 21-item structure [22]. The Turkish version is also a valid tool for assessing hip OA [23].

The Mini-OAKHQoL is a 20-item questionnaire designed to evaluate the quality of life in individuals with hip and/or knee OA. It captures patient experiences over the past month across five domains—physical activity, emotional well-being, pain, social relationships, and social participation—and includes three standalone questions regarding sexual health, work life, and concerns about becoming dependent [24]. The Turkish adaptation has demonstrated sufficient validity and reliability in evaluating the quality of life among individuals with hip and/or knee osteoarthritis [25].

*Translation and Cross-cultural adaptation:* The French/English version of the questionnaire was translated into Turkish and culturally adapted using a standardized forward and backward translation method [26]. Three physicians originally translated the French/English Flare-OA into Turkish. After achieving an agreement on the coherence and sufficiency of the meanings of the various translations, two linguists, one of whom is a native speaker, translated the Turkish version back into English and French. The final form of the Turkish translations was then agreed upon by an expert committee consisting of three rheumatologists and two translators (Supplementary File-1 and 2). The Turkish version was reviewed by an expert panel to ensure clarity, conceptual equivalence, and cultural relevance, followed by pretesting with a sample of ten patients.

*Content and face validities:* The pre-final version of the scale was evaluated by a panel of three clinicians specializing in rheumatology and physical medicine, one of whom had advanced proficiency in both French and English. The items were assessed to ensure they were relevant to osteoarthritis flare characteristics. Additionally, face validity was assessed through cognitive interviews with ten patients, focusing on the clarity, ease of understanding, and practical usability of the translated scale.

*Construct validity:* Construct validity was evaluated with convergent and divergent validity. Convergent validity was examined by correlating Flare-OA scores with HOOS-PS, KOOS, and Mini-OAKHQOL scores.

*Reliability*: Internal consistency was assessed using Cronbach’s alpha coefficient [27]. To evaluate test-retest reliability, participants were invited to complete the Flare-OA questionnaire again two weeks after the initial administration. During this interval, no therapeutic intervention was applied, and patients were contacted by mail for follow-up. Reproducibility was determined among those who reported no change in their flare status. The intraclass correlation coefficient (ICC) was computed based on the total score, and the standard error of measurement (SEM) was calculated using the formula: SD × √(1 − ICC), to contextualize the difference between the flare and non-flare groups [28].

*Sensitivity to change:* Sensitivity to change was assessed among participants who reported either improvement or worsening in response to the flare question at the second evaluation. The Standardized Response Mean (SRM) was used to quantify change, with thresholds of ≥ 0.20, ≥ 0.50, and ≥ 0.80 corresponding to small, moderate, and large levels of change, respectively [29].

*Floor and ceiling effects:* Floor and ceiling effects, which may limit the responsiveness of a measurement instrument, were examined by analyzing the distribution of responses at both extremes of the scale. These effects were deemed to exist if more than 15% of participants selected the minimum (0) or maximum (10) score for a given item [30].

### Statistical analysis

Descriptive statistics were reported as mean ± standard deviation (SD), median and range (minimum–maximum), and frequencies for categorical data. For construct validity, a confirmatory factor analysis examined the adequation of the Turkish version to the original model using goodness of fit indices: root mean square error of approximation (RMSEA ≤ 0.06), standardized root mean residual (SRMR ≤ 0.08) and comparative fit index (CFI ≥ 0.95). Convergent and divergent validity were evaluated using Pearson correlation coefficients. Descriptive analyses were conducted to explore the frequency distribution of individual Flare-OA items. Internal consistency was assessed via Cronbach’s alpha, with a threshold of > 0.70 indicating acceptable reliability. Test-retest reliability was quantified using intraclass correlation coefficients (ICCs), where values exceeding 0.75 were interpreted as demonstrating good reliability. To complete the classical test theory approach, item response theory was conducted applying Rasch analysis with the 4 final partial credit models of 16-item version of Flare OA obtained in the French/English study at each domain, with the same criteria for checking assumption: unidimensionality, local dependency, invariance, and evaluating goodness-of-fit used in the French/English version [18]. *P* <.05 was considered statistically significant. Statistical analysis was performed using the SAS v9.4 (Cary, NC, USA) and using RUMM2030 software (RUMM Laboratory Pty, Ltd, Duncraig, WA) for Rasch model analysis.

## Results

**Fig. 1:**
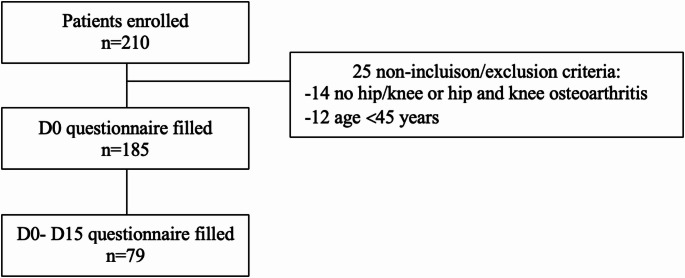
Flow chart of the included patients

The demographic and clinical parameters are presented in Table 2. Initially, 210 patients with knee or hip OA were evaluated and 25 did not meet the inclusion requirements and were therefore excluded. The research comprised 185 patients, 79 of whom were re-evaluated at week two (Fig. 1). The study included 185 participants, of whom 71.9% were women, with a mean age of 63.2 years (SD:9.1). Among them, 160 (86.5%) were diagnosed with knee OA, 25 (13.5%) with hip OA, and 70 (37.8%) reported a flare-up of symptoms in the previous four weeks.

**Table 2:** Description of included patients at baseline

	Flare of osteoarthritis		
	No (*n* = 115)	Yes (*n* = 70)	Total (*n* = 185)
Gender			
Female	75 (65.25)	58 (82.9%)	133 (71.9%)
Male	40 (34.8%)	12 (17.1%)	52 (28.1%)
Age	61.4 (8.36)	66 (9.62)	63.2 (9.1)
Joint affected by OA			
Knee	96 (83.5%)	64 (91.4%)	160 (86.5%)
Knee, left	69 (60%)	54 (77.1%)	123 (66.5%)
Knee, right	66 (57.4%)	54 (77.1%)	120 (64.9%)
Hip	19 (16.5%)	6 (8.6%)	25 (13.5%)
Hip left	11 (9.6%)	5 (7.1%)	16 (8.6%)
Hip, right	12 (10.4%)	2 (2.9%)	14 (7.6%)
Duration of flare	–	9 (7.73)	
Have a flare at D0	–	30 (42.9%)	30 (16.2%)
Prosthesis			
None	109 (94.8%)	66 (94.3%)	175 (94.6%)
Yes	6 (5.3%)	4 (5.7%)	10 (5.4%)
Medication for OA			
No	70 (60.9%)	23 (32.9%)	93 (50.3%)
Yes	45 (39.1%)	47 (67.1%)	92 (49.7%)

*OA* Osteoarthritis,* D0* First survey

Data are presented as mean± SD or n (%)

The pre-final Turkish version of the Flare-OA was evaluated for content validity. Cognitive interviews were conducted with ten patients to assess their understanding of each item. All patients reported that the items were clear and readily understandable, and they thought the scale to be practical and applicable. Consequently, there was no requirement for any additional cultural adjustments. Hence, the scale demonstrated suitable face and content validities, and the original meaning of the items remained unchanged.

There was no missing data in any item, and all questions were answered by all patients. While there is a floor effect in all items, only 1 item showed a ceiling effect (I32, 16.2%) (Floor and ceiling effects at both item and subscale levels for the Turkish version of the Flare-OA are provided in Supplementary File-3).

### Construct validity by confirmatory factor analysis

Cronbach’s alpha coefficient was 0.987 (95% CI0.984–0.990) for the 33-item and 0.972 (95% Cl 0.966–0.978) for 16-item. Cronbach’s alpha (33-item) for each domain was 0.93 (95% Cl 0.924–0.952) for painful, 0.897 (95% Cl 0.863–0.923) for swelling, 0.97 (95% Cl 0.964–0.980) for stiffness, 0.97 (95% Cl 0.973–0.982) for consequences of symptoms, and 0.97 (95% Cl 0.966–0.978) for psychological aspects.

Cronbach’s alpha (16-item) for each domain, were 0.909 (95% Cl 0.885–0.928) for painful, 0.973 (95% Cl 0.964–0.980) for stiffness, 0.924 (95% Cl 0.905–0.941) for consequences of symptoms, and 0.945 (95% Cl 0.931–0.956) for psychological aspects. Since Cronbach’s alpha can be calculated for those with at least two items and there is only 1 item in the swelling part in 16 items, Cronbach’s alpha could not be calculated for swelling.

In all versions, Flare-OA scores showed a significant correlation with both KOOS and mini-OAKHQOL scores (Table 3).

**Table 3:** Pearson’s correlation coefficients of the Flare-OA questionnaire with the other parameters for construct validity

Parameters	33-item (*r*)						Total	Pain	Swelling	Stiffness	16-item (*r*)	
	Total	Pain	Swelling	Stiffness	Consequences of symptoms	Psychological aspects					Consequences of symptoms	Psychological aspects
HOOS (0-100) (*n* = 25)	0.69	0.64	0.54	0.64	0.69	0.62	0.67	0.58		0.64	0.67	0.62
KOOS (0-100) (*n* = 160)												
The activity of daily living	– 0.83*	– 0.74	– 0.56	– 0.66	– 0.82*	– 0.82*	– 0.83*	– 0.73	– 0.53	– 0.66	– 0.83*	– 0.83*
Other symptoms	– 0.79*	– 0.7	– 0.59	– 0.65	– 0.76*	– 0.78*	– 0.78*	– 0.67	– 0.56	– 0.65	– 0.76	– 0.78*
Pain KOOS	– 0.83*	– 0.75	– 0.58	– 0.66	– 0.82*	– 0.81*	– 0.83*	– 0.72	– 0.55	– 0.66	– 0.82*	– 0.84*
Quality of life	– 0.78*	– 0.69*	– 0.46	– 0.61*	– 0.77*	– 0.79*	– 0.79*	– 0.67*	– 0.43	– 0.61*	– 0.78*	– 0.82*
Sport and recreation	– 0.69*	– 0.64*	– 0.38	– 0.55	– 0.69*	– 0.66*	– 0.70*	– 0.60*	– 0.36	– 0.55	– 0.72*	– 0.72*
Mini-OAKHQOL (0-100) (*n* = 185)												
Mental health	– 0.82*	– 0.71	– 0.54	– 0.67	– 0.78*	– 0.84*	– 0.81*	– 0.70	– 0.53	– 0.67	– 0.77*	– 0.83*
Pain OAKHQOL	– 0.86*	– 0.790	– 0.56	– 0.73	– 0.84*	– 0.85*	– 0.87*	– 0.77*	– 0.54	– 0.73	– 0.84*	– 0.87*
Physical activities	– 0.84*	– 0.76	– 0.54	– 0.67	– 0.83*	– 0.82*	– 0.85*	– 0.73	– 0.52	– 0.67	– 0.85*	– 0.86*
Social functioning	– 0.07	– 0.06	– 0.02	– 0.03	– 0.09	– 0.06	– 0.07	– 0.05	– 0.0	– 0.03	– 0.11	– 0.07
Social support	0.05	0.05	0.01	0.06	0.05	0.04	0.05	0.03	0.0	0.06	0.07	0.05

*indicates p <.05, * HOOS* Hip Disability and Osteoarthritis Outcome Score,* KOOS* Knee Injury and Osteoarthritis Outcome Score,* Mini-OAKHQOL* Mini Osteoarthritis Knee and Hip Quality of Life Questionnaire

### Discriminant validity

Discriminant validity was supported by a statistically significant difference in scores, with a mean value of 36.2 (95% CI29.9–42.6, SEM:8, *p* <.0001) for 33-item between patients with and without flare [Painful 38.8 (95% CI 32.8–44.8, SEM:9.6, *p* <.0001), swelling 24.4 (95% CI 16.3–32.5, SEM:11, *p* <.0001), stiffness 42.9 (95% CI 4.7–51.1, SEM:15.7, *p* <.0001), consequences of symptoms 35.5 (95% CI 28.6–42.3, SEM:9.5, *p* <.0001), psychological aspects 36.8 (95% CI 29.1–44.5, SEM:11.2, *p* <.0001)].

Discriminant validity was supported by a statistically significant difference in scores, with a mean value of 36.2 36.7 (95% CI 30.3–43, SEM: 8.1, *p* <.0001), for 16-item between patients with and without flare [Painful 37.6 (95% CI 30.9–44.4, SEM:9.2, *p* <.0001), swelling 27.2 (95% CI 18.4–36, SEM:11, *p* <.0001), stiffness 42.9 (95% CI 34.7–51.1, SEM:15.7, *p* <.0001), consequences of symptoms 35.3 (95% CI 28-42.6, SEM:9.8, *p* <.0001), psychological aspects 36.9 (95% CI 29.2–44.7, SEM:11.4, *p* <.0001)].

### Reliability

Test-retest was assessed for 79 patients at an interval of two weeks. ICC was above the threshold of 0.70 [(ICC 0.913, 95% Cl 0.868–0.943) and (ICC 0.912, 95% Cl 0.866–0.943), for 33 and 16 items, respectively)], indicating good test-retest reliability.

ICC scores were as follows for 33 and 16 items, respectively, according to domains: Painful 0.864 (95% CI 0.796–0.911) and 0.878 (95% CI 0.816–0.920); swelling 0.785 (95% CI 0.683–0.857) and 0.782 (95% CI 0.661–0.864); stiffness 0.730 (95% CI 0.609–0.819) and 0.730 (95% CI 0.609–0.819); consequences of symptoms 0.887 (95% CI 0.830–0.926) and 0.888 (95% CI 0.830–0.927); psychological aspects 0.886 (95% CI 0.827–0.925) and 0.879 (95% CI 0.818–0.921).

### Sensitivity to change

Of the 34 patients who described a flare at baseline, 9 reported no flare at week two. Sensitivity to change with flare improvement [SRM 1.2 (95% CI 0.6–1.7) and SRM 1 (95% CI 0.5–1.5), for 33- and 16-item, respectively ] over the period.

SRM scores were as follows for 33- and 16-item, respectively, according to domains: Painful 0.9 (95% Cl 0.6–1.7) and 0.8 (95% Cl 0.2–1.6); swelling 0.1 (95% Cl -0.7-0.8) and 0.2 (95% CI -0.6-1.0); stiffness 0.1 (95% CI -0.7-0.8) and 0.1 (95% CI– 0.7–0.8); consequences of symptoms 1.1 (95% CI 0.6–1.6) and 0.8 (95% CI 0.3–1.2); psychological aspects 0.9 (95% CI 0.3–1.8) and 0.9 (95% CI 0.4–1.7).

### Rasch analysis

It revealed a local dependency between 2 items of the Consequences of symptoms domain: “I had to shorten my walking distance” and “I had more difficulty getting in or out of a car”; and between 2 items of Psychological aspects: “I needed to rest (e.g., lie down or sit) to prevent my pain” and “I needed to change the way I performed daily activities (e.g., sitting instead of standing when getting dressed or preparing food) to prevent my pain,” not identified in the French/English sample. This was solved by creating 2 super-item scores, combining response modalities of each two items into one scale. After this update, the 4 models for each domain in Turkish demonstrated good properties: well-ordered thresholds on the latent trait with recoded items of the French/English sample, unidimensionality respected with less than 5% of t-test comparisons significant, no more local dependency with a residual correlation 0.3 point above the average of all items residual correlations, and invariance respected with no significant DIF by age, sex, and joint. Goodness of fit of the 4 models was good, with p global item trait interaction Khi-2 no significant after bonferroni adjusted correction, a range of individual item-fit residual within ± 2.5 (between − 1.89 and 1.84), and a good reliability measured with Person Separation Index > 0.7 except for the Pain domain (PSI = 0.68). The person-item threshold distribution showed, overall, a good match between the location of items and of persons for each domain. Table 4 provides the raw sum score within each four domains that was summed to calculate the Rasch composite score in the Turkish sample (see Supplementary File-4 to calculate the Rasch composite score of the Turkish 16-item version of Flare-OA).

**Table 4:** Conversion table from Raw scores in each dimension to a linear measure—values estimated by Rasch model in Turkish sample

Raw sum score	Linear measure				Raw sum score	Linear measure		
	Logit scale		0-10 scale			Logit scale	0-10 scale	
Pain**	Knee	Hip	Knee	Hip	Impact of symptoms			
0	− 4.06	− 4.02	0.00	0.00	0	− 3.40		0.00
1	− 2.83	− 2.63	1.26	1.44	1	− 2.80		0.90
2	− 1.78	− 1.62	2.34	2.50	2	− 2.22		1.76
3	− 0.87	− 0.87	3.27	3.27	3	− 1.65		2.60
4	− 0.24	− 0.31	3.93	3.85	4	− 0.92		3.69
5	0.24	0.15	4.41	4.32	5	− 0.11		4.90
6	0.67	0.57	4.86	4.76	6	0.59		5.94
7	1.16	1.03	5.36	5.25	7	1.18		6.83
8	1.83	1.68	6.05	5.92	8	1.76		7.69
9	3.14	2.99	7.40	7.27	9	2.46		8.73
10	5.68	5.62	10.00	10.00	10	3.31		10.00
Stiffness					Psychological aspects			
0	− 8.93		0.00		0	− 3.99	0.00	
1	− 6.06		1.93		1	− 3.30	0.83	
2	− 3.80		3.44		2	− 2.60	1.67	
3	− 2.01		4.64		3	− 1.90	2.51	
4	− 0.87		5.40		4	− 1.04	3.54	
5	− 0.03		5.97		5	− 0.39	4.33	
6	0.51		6.33		6	0.11	4.92	
7	0.92		6.60		7	0.57	5.48	
8	1.31		6.86		8	1.08	6.10	
9	1.74		7.15		9	1.75	6.90	
10	2.28		7.51		10	2.77	8.13	
11	2.99		7.99		11	4.33	10.00	
12	4.13		8.76					
13	5.99		10.00					

* Swelling dimension (knee) has only one item (FL5) and maintains the raw score

** Pain score calculation differs by joint because of a split for item 4 “My pain disrupted my sleep more than usual” to take into account uniform DIF of item 4 by joint

## Discussion

The current study cross-culturally adapted the Flare-OA scales (33-item and 16-item) into Turkish in patients with knee or hip OA and found them to be understandable and well accepted by participants. The scale exhibited strong internal consistency and test-retest reliability, suggesting that it is a stable and dependable instrument for measuring flares. The content validity, confirmed by expert reviews, and the construct validity, supported by correlations with established measures of OA severity and patient-reported outcomes, further affirm the scale’s applicability. These findings indicate that the Turkish versions of the scale can effectively capture the episodic nature of symptoms in patients with knee or hip OA over the past 4 weeks before clinical visit, providing clinicians and researchers with a valuable tool for assessment and monitoring.

The qualitative research conducted in this study demonstrates that flare is more complex than the presence or absence of pain. A more comprehensive assessment approach was required to accurately evaluate flare, as it was necessary to capture the complete spectrum of components that define a flare. A major limitation in the field has been the lack of a standardized definition of OA flare and the absence of a validated tool that adequately incorporates the patient perspective. As Parry et al. mentioned in the recent review of defining flares in knee OA, many studies have used different terms (flare, flare-up, worsening of symptoms) and definitions to describe OA flares. Most studies have used an increase in pain over ‘usual’ or ‘baseline’ intensity as a flare definition [12]. Aside from pain, swelling, morning stiffness, alterations in mood, psychological conditions including anxiety and depression, and changes in daily activities due to symptoms are significant observations and outcomes of OA [11].

Of our patients, 86.5% were diagnosed with knee OA, 13.5% had hip OA, and 37.8% of the patients reported a flare of their hip or knee OA within the last four weeks. In the Flare OA validation research, the patient group consisted of 70.4% females, 86% had knee OA, and 13.3% had hip OA. However, a somewhat higher percentage of patients (64.8%) experienced a flare in the past four weeks compared to our study [14]. Internal consistency of the Turkish Flare OA scales was high with a Cronbach’s alpha of 0.98 and 0.97 for 33- and 16-items, respectively, very close to the Cronbach’s alpha of the original Flare-OA, which was 0.96 [14]. These findings indicate that all versions of the Turkish Flare-OA questionnaire demonstrate internal consistency comparable to the original version, thereby supporting the reliability of the Turkish adaptation. Moreover, the Rasch analysis demonstrated a good fit of the scale, allowing to calculate a Rasch score with interval scale property. A further analysis of English/French/Turkish versions demonstrated invariance across versions, in favour of a robust measurement scale.

The ability of a questionnaire to differentiate between patients reporting a flare and those who do not is indicative of its capacity to measure and characterize a flare in OA. This is demonstrated by a significant difference of over 30 points in the aggregate group scores. The Flare-OA questionnaire is capable of distinguishing and quantifying various flare states, at least at the population level, as evidenced by a SEM of 8.

The activity of daily living, other symptoms, pain, quality of life, and sport and recreation with KOOS parameters had good correlations with all Flare OA questions, suggesting a good convergent validity. KOOS-pain scores had the highest correlation with all Flare-OA scales, and in the original Flare-OA validation study, the highest correlation was also with KOOS-pain scores (*r*=-.072) [14]. The correlation with HOOS scores in evaluating the hip was not significant. This may be due to the small number of patients with hip OA participating in this study.

Quality of life is a significant concern that is adversely impacted by symptoms and flares in those with OA [31]. A meta-analysis revealed that individuals with OA exhibited significantly lower scores in all domains of the SF-36 encompassing physical functioning, role limitations due to physical and emotional problems, pain, general health, vitality, social functioning, and mental well-being when compared to healthy controls. This difference was particularly significant in the dimension of physical role function [32]. The Mini-OAKHQOL is also a valid scale assessing the quality of life in patients with lower extremity OA, and the subdomains of mental health, pain, and physical activities showed a high correlation with all versions of flare-OA.

Item 32 (“I couldn’t do anything to help relieve the pain”) showed a moderate floor effect (28.6%) and a slight ceiling effect (7.6%). Although these values generally remain within an acceptable range (< 30%), the higher floor effect suggests that a significant portion of participants strongly disagreed with this item, indicating that it may have limited discriminatory power in detecting mild to moderate symptoms. In future longitudinal applications or intervention studies, the utility and sensitivity of item 32 may be affected.

One of the limitations of this study is the exclusion of individuals diagnosed with both knee and hip OA. This decision was made to ensure that the instrument could assess flare characteristics specific to a single joint site. As a result, patients experiencing symptoms in both joints may not be fully represented. Nonetheless, the questionnaire was constructed to be joint-neutral in content, and item-level acceptability was high across both knee and hip OA groups. Additionally, the current analysis did not include an evaluation of the scale’s responsiveness to clinical change. Future research involving larger, more heterogeneous samples is warranted to confirm these findings and to investigate the scale’s sensitivity to treatment effects over time. The last point is that the relatively small number of patients with hip OA may limit the robustness and generalizability of subgroup-specific analyses. However, it is important to note that this proportion is consistent with the original Flare-OA validation study, in which hip OA also constituted approximately 13% of the sample. This reflects the expected distribution of knee and hip OA in the general population [14]. The relatively weak correlations observed with HOOS are likely attributable to the small hip OA sample size. Nonetheless, further validation in largerand more diverse populations remains essential to strengthen subgroup-specific conclusions.

This study contributed significantly to the current literature by defining OA flares and translating the Flare-OA questionnaire into Turkish. Previous research has evaluated the psychometric features of the Flare-OA scale for French- and English-speaking populations, but this is the first to translate, culturally adapt, and psychometrically verify both the 33-item and short 16-item versions of the Flare-OA in Turkish.

In conclusion, the Turkish versions of the Flare-OA questionnaires (33-item and 16-item forms) demonstrated strong reliability, good validity, and practical applicability in assessing disease flares in patients with knee and hip OA. The availability of a 16-item form is particularly valuable for use in routine clinical practice and research settings. Although the results are promising, the relatively small sample size of the hip OA subgroup is a limitation and highlights the need for further validation in larger and more diverse populations. Overall, the scale provides a comprehensive and feasible tool to capture all key dimensions of OA flares in the Turkish population.

## Electronic supplementary material

Below is the link to the electronic supplementary material.

Supplementary Material 1.

Supplementary Material 2.

Supplementary Material 3.

Supplementary Material 4.

Supplementary Material 5.

## Data Availability

Data supporting the findings of this study are available from the corresponding author upon reasonable request. Data sharing complies with institutional and ethical guidelines.

## Declarations

### Note on related congress abstracts and publication

Accepted at the ACR Convergence 2023 Duruöz M, Gezer H, Epstein J, Soudant M, Guillemin F. Cross-cultural Adaptation, Reduction, and Validation of the Turkish Versions of the Flare-OA Questionnaire for Hip and Knee Osteoarthritis [abstract]. Arthritis Rheumatol. 2023; 75 (suppl 9). https://acrabstracts.org/abstract/cross-cultural-adaptation-reduction-and-validation-of-the-turkish-versions-of-the-flare-oa-questionnaire-for-hip-and-knee-osteoarthritis/.
