# Supplementary material for: Validity and reliability of the Flare-OA scale for hip and knee osteoarthritis in a Turkish population: item reduction with Rasch analysis
Source: Rheumatol Int. 2025 Jul 3;45(7):163. doi: 10.1007/s00296-025-05914-3 (PMC12226661; doi:10.1007/s00296-025-05914-3)
Supplement: Supplementary file 1 — Supplementary Material 1. [file 296_2025_5914_MOESM1_ESM.docx]

**Supplementary 3**

Table. Item-based and subscale-based floor effect and ceiling effect of the Turkish version of Flare-OA

| **Items** | **Floor effect (%)*** | **Ceiling effect (%)**** |
| --- | --- | --- |
| **Painful** |  |  |
| I1 | 54 (29.2%) | 8 (4.3%) |
| I2 | 55 (29.7%) | 12 (6.5%) |
| I3 | 40 (21.6% | 10 (5.4%) |
| I4 | 41 (22.2%) | 8 (4.3%) |
| I5 | 46 (24.9%) | 12 (6.5%) |
| I6 | 56 (30.3%) | 7 (3.8%) |
| **Swelling** |  |  |
| I7 | 89 (48.1%) | 4 (2.2%) |
| I8 | 80 (43.2%) | 3 (1.6%) |
| **Stiffness** |  |  |
| I9 | 48 (25.9%) | 17 (9.2%) |
| I10 | 44 (23.8%) | 14 (7.6%) |
| **Consequences of symptoms (sleep, concentration, activity, need for help, walking)** |  |  |
| I11 | 56 (30.3%) | 9 (4.9%) |
| I12 | 35 (18.9%) | 16 (8.6%) |
| I13 | 36 (19.5%) | 10 (5.4%) |
| I14 | 69 (37.3%) | 3 (1.6%) |
| I15 | 57 (30.8%) | 8 (4.3%) |
| I16 | 65 (35.1%) | 6 (3.2%) |
| I17 | 53 (28.6%) | 10 (5.4%) |
| I18 | 66 (35.7%) | 6 (3.2%) |
| I19 | 35 (18.9%) | 24 (13%) |
| I20 | 34 (18.4%) | 19 (10.3%) |
| I21 | 32 (17.3%) | 24 (13%) |
| I22 | 41 (22.2%) | 14 (7.6%) |
| I23 | 30 (16.2%) | 25 (13.5%) |
| I24 | 31 (16.8%) | 27 (14.6%) |
| **Psychological aspects** |  |  |
| I25 | 47 (25.4%) | 20 (10.8%) |
| I26 | 56 (30.3%) | 15 (8.1%) |
| I27 | 53 (28.6%) | 14 (7.6%) |
| I28 | 54 (29.2%) | 13 (7%) |
| I29 | 59 (31.9%) | 11 (5.9%) |
| I30 | 31 (16.8%) | 24 (13%) |
| I31 | 30 (16.2%) | 30 (16.2%) |
| I32 | 53 (28.6%) | 14 (7.6%) |
| I33 | 33 (17.8%) | 25 (13.5%) |

* Proportion of the sample at the maximum scale range >15%

** Proportion of the sample at the minimum scale range >15%
