# Supplementary material for: Validity and reliability of the Flare-OA scale for hip and knee osteoarthritis in a Turkish population: item reduction with Rasch analysis
Source: Rheumatol Int. 2025 Jul 3;45(7):163. doi: 10.1007/s00296-025-05914-3 (PMC12226661; doi:10.1007/s00296-025-05914-3)
Supplement: Supplementary file 3 — Supplementary Material 3. [file 296_2025_5914_MOESM3_ESM.docx]

**Supplementary 1 OA HASTALIK ALEVLENME ANKETİ-33**

**Ağrı:** *Son dört hafta boyunca*

|  | **Kesinlikle**  **değil**  **0** | **1** | **2** | **3** | **4** | **5** | **6** | **7** | **8** | **9** | **Kesinlikle 10** |
| --- | --- | --- | --- | --- | --- | --- | --- | --- | --- | --- | --- |
| 1. Hissettiğim ağrılar her zamankinden farklıydı. |  |  |  |  |  |  |  |  |  |  |  |
| 1. Ağrım her zamanki ağrımdan daha şiddetliydi. |  |  |  |  |  |  |  |  |  |  |  |
| 1. Dinlendiğimde ağrı geçmedi. |  |  |  |  |  |  |  |  |  |  |  |
| 1. Bir süre hareketsiz kaldıktan sonra hareket ettiğimde, ağrı ya da rahatsızlık geçmedi. |  |  |  |  |  |  |  |  |  |  |  |
| 1. Gece ağrım oldu. |  |  |  |  |  |  |  |  |  |  |  |
| 1. Ağrım her zamankinden daha kalıcıydı. |  |  |  |  |  |  |  |  |  |  |  |

**Şişlik:** *Son dört hafta boyunca*

|  | **Kesinlikle**  **değil**  **0** | **1** | **2** | **3** | **4** | **5** | **6** | **7** | **8** | **9** | **Kesinlikle 10** |
| --- | --- | --- | --- | --- | --- | --- | --- | --- | --- | --- | --- |
| 1. Dizim her zamankinden daha fazla şişti. |  |  |  |  |  |  |  |  |  |  |  |
| 1. Dizimde sıvı varmış gibi hissettim. |  |  |  |  |  |  |  |  |  |  |  |

**Tutukluk:** *Son dört hafta boyunca*

|  | **Kesinlikle**  **değil**  **0** | **1** | **2** | **3** | **4** | **5** | **6** | **7** | **8** | **9** | **Kesinlikle 10** |
| --- | --- | --- | --- | --- | --- | --- | --- | --- | --- | --- | --- |
| 1. Eklemimi (diz veya kalça) hareket ettirirken her zamankinden daha fazla zorlandım. |  |  |  |  |  |  |  |  |  |  |  |
| 1. Eklemimde her zamankinden daha fazla tutukluk hissettim. |  |  |  |  |  |  |  |  |  |  |  |

**Semptomların sonuçları:** *Son dört hafta boyunca*

**(Uyku, konsantrasyon, aktivite, yardım ihtiyacı, yürüme)**

|  | **Kesinlikle**  **değil**  **0** | **1** | **2** | **3** | **4** | **5** | **6** | **7** | **8** | **9** | **Kesinlikle 10** |
| --- | --- | --- | --- | --- | --- | --- | --- | --- | --- | --- | --- |
| 1. Ağrım uykumu her zamankinden daha fazla bozdu. |  |  |  |  |  |  |  |  |  |  |  |
| 1. Ağrım günlük işlerimden bazılarını yapmamı engelledi. |  |  |  |  |  |  |  |  |  |  |  |
| 1. Ağrım yapmak zorunda olduğum işe konsantre olmamı engelledi. |  |  |  |  |  |  |  |  |  |  |  |
| 1. Eklemimi sabitlemek zorunda kaldım (Örn: diz desteği veya bandaj). |  |  |  |  |  |  |  |  |  |  |  |
| 1. Her zamankinden daha fazla ağrı kesici kullanmaya ihtiyacım oldu. |  |  |  |  |  |  |  |  |  |  |  |
| 1. Diz veya kalça eklemime her zamankinden daha fazla buz veya soğuk uygulama ihtiyacım oldu. |  |  |  |  |  |  |  |  |  |  |  |
| 1. Günlük işlerimi yapmak için yakınlarımdan her zamankinden daha fazla yardım istemek zorunda kaldım. |  |  |  |  |  |  |  |  |  |  |  |
| 1. Yürümek ya da ayakta durabilmek için her zamankinden daha fazla yardıma ihtiyacım oldu (Örn: Baston ya da koltuk değneği kullanmak, parmaklıklara yaslanmak). |  |  |  |  |  |  |  |  |  |  |  |
| 1. Günlük hayatımın olumsuz yönde değiştiğini hissediyorum. |  |  |  |  |  |  |  |  |  |  |  |
| 1. Hareketlerimde daha fazla kısıtlanma veya azalma hissettim. |  |  |  |  |  |  |  |  |  |  |  |
| 1. Yürüme mesafemi azaltmak zorunda kaldım. |  |  |  |  |  |  |  |  |  |  |  |
| 1. Günlük programımı değiştirmek zorunda kaldım. |  |  |  |  |  |  |  |  |  |  |  |
| 1. Bozuk zeminde yürürken daha fazla zorlandım. |  |  |  |  |  |  |  |  |  |  |  |
| 1. Arabaya binerken veya inerken daha fazla zorlandım. |  |  |  |  |  |  |  |  |  |  |  |

**Psikolojik durum:** *Son dört hafta boyunca*

**(Duygu durumu, sıkıntı, hayal kırıklığı)**

|  | **Kesinlikle**  **değil**  **0** | **1** | **2** | **3** | **4** | **5** | **6** | **7** | **8** | **9** | **Kesinlikle 10** |
| --- | --- | --- | --- | --- | --- | --- | --- | --- | --- | --- | --- |
| 1. Ağrım duygu durumumu her zamankinden daha fazla etkiledi. |  |  |  |  |  |  |  |  |  |  |  |
| 1. **Kendimi her zamankinden daha fazla depresyonda hissettim.** |  |  |  |  |  |  |  |  |  |  |  |
| 1. Günlük işlerimdeki kısıtlılıktan dolayı hayal kırıklığına uğramış hissettim. |  |  |  |  |  |  |  |  |  |  |  |
| 1. **Her zamankinden daha fazla endişeli hissettim.** |  |  |  |  |  |  |  |  |  |  |  |
| 1. **Çaresiz hissettim.** |  |  |  |  |  |  |  |  |  |  |  |
| 1. Ağrımı önlemek için belirli hareketler veya aktivitelerden kaçınmam gerekti. |  |  |  |  |  |  |  |  |  |  |  |
| 1. Ağrı olmaması için dinlenmeye ihtiyaç duydum (Örn: Uzanmak veya oturmak). |  |  |  |  |  |  |  |  |  |  |  |
| 1. Ağrı olmaması için yapabileceğim hiçbir şey yoktu. |  |  |  |  |  |  |  |  |  |  |  |
| 1. Ağrı olmaması için günlük işlerimi yapma şeklini değiştirmem gerekti (Örn: Giyinirken veya yemek hazırlarken ayakta durmak yerine oturmak). |  |  |  |  |  |  |  |  |  |  |  |
