# Supplementary material for: Validity and reliability of the Flare-OA scale for hip and knee osteoarthritis in a Turkish population: item reduction with Rasch analysis
Source: Rheumatol Int. 2025 Jul 3;45(7):163. doi: 10.1007/s00296-025-05914-3 (PMC12226661; doi:10.1007/s00296-025-05914-3)
Supplement: Supplementary file 4 — Supplementary Material 4. [file 296_2025_5914_MOESM4_ESM.docx]

**Supplementary 4: How to calculate the composite score of Turkish version FLARE-OA-16?**

1. **Recoding of items**

**Table S1 – Recoding of items in Rasch model for Turkish sample**

| **Dimensions** | **Item Number** | **Coding** | **Recoding** |
| --- | --- | --- | --- |
| **Pain** | 1 | 0 to 4 | 0 |
|  |  | 5 to 7 | 1 |
|  |  | 8 to 10 | 2 |
|  | 2 | 0 | 0 |
|  |  | 1 to 3 | 1 |
|  |  | 4 to 6 | 2 |
|  |  | 7 to 10 | 3 |
|  | 3 | 0 | 0 |
|  |  | 1 to 4 | 1 |
|  |  | 5 to 8 | 2 |
|  |  | 9 to 10 | 3 |
|  | 4 | 0 | 0 |
|  |  | 1 to 5 | 1 |
|  |  | 6 to 10 | 2 |
|  | Super-item 1+3* |  | 0 to 5 |
| **Swelling** | 5** | 0 to 10 | 0 to 10 |
| **Stiffness** | 6 | 0 | 0 |
|  |  | 1 to 3 | 1 |
|  |  | 4 | 2 |
|  |  | 5 to 6 | 3 |
|  |  | 7 | 4 |
|  |  | 8 | 5 |
|  |  | 9 to 10 | 6 |
|  | 7 | 0 | 0 |
|  |  | 1 to 3 | 1 |
|  |  | 4 | 2 |
|  |  | 5 | 3 |
|  |  | 6 | 4 |
|  |  | 7 | 5 |
|  |  | 8 | 6 |
|  |  | 9 to 10 | 7 |
| **Consequences of symptoms**  **Psychological aspects** | 8 | 0 | 0 |
|  |  | 1 to 3 | 1 |
|  |  | 4 to 6 | 2 |
|  |  | 7 to 10 | 3 |
|  | 9 | 0 | 0 |
|  |  | 1 to 5 | 1 |
|  |  | 6 to 10 | 2 |
|  | 10 | 0 | 0 |
|  |  | 1 to 5 | 1 |
|  |  | 6 to 10 | 2 |
|  |  | 0 | 0 |
|  | 11 | 1 to 2 | 1 |
|  |  | 3 to 7 | 2 |
|  |  | 8 to 10 | 3 |
|  | Super-item 10+11*** |  | 0 to 5 |
| **Psychological aspects** | 12 | 0 | 0 |
|  |  | 1 to 5 | 1 |
|  |  | 6 to 10 | 2 |
|  | 13 | 0 | 0 |
|  |  | 1 to 3 | 1 |
|  |  | 4 to 6 | 2 |
|  |  | 7 to 10 | 3 |
|  | 14 | 0 | 0 |
|  |  | 1 to 4 | 1 |
|  |  | 5 to 10 | 2 |
|  | 15 | 0 | 0 |
|  |  | 1 to 6 | 1 |
|  |  | 7 to 10 | 2 |
|  | 16 | 0 | 0 |
|  |  | 1 to 3 | 1 |
|  |  | 4 to 10 | 2 |
|  | Super-item 14+16**** |  | 0 to 4 |

* Recoding items 1 and 3 (indicated in their respective lines in grey police on the table) are combined by sum into a single super-item 1+3 because of local dependency between each two items, which varies from 0 (0+0) to 5 (2+3).

** If patient has osteoarthritis of the hip, pre code item 5 to 0 (Not at all), whatever the answer to this item as this item only concerns patients with osteoarthritis of the knee.

*** Recoding items 10 and 11 (indicated in their respective lines in grey police on the table) are combined by sum into a single super-item 10+11 because of local dependency between each two items, which varies from 0 (0+0) to 5 (2+3).

**** Recoding items 14 and 16 (indicated in their respective lines in grey police on the table) are combined by sum into a single super-item 14+16 because of local dependency between each two items, which varies from 0 (0+0) to 4 (2+2).

1. **Calculation of Rasch scores of four domains of Turkish version Flare-OA-16 on a 0-10 linear scale**

Rasch scores of four domains of Flare-OA-16 are calculated summing up recoded items within each domain (raw sum score) and then using Rasch conversion table (table, column 0-10 scale) obtained from a partial credit model to have a 0-10 linear measure.

1. **Calculation of Rasch composite score of Turkish version FLARE-OA-16**

Rasch composite score of Turkish version FLARE-OA-16 is calculated by averaging four Rasch 0 to 10 scales for hip OA and averaging the four Rasch 0 to10 scales plus item 5 (range 0 to 10) for knee OA (taking into account knee item only for knee osteoarthritis) with the following formula by joint OA:

**FOACS_RASCH _knee OA_ =** $\frac{\mathbf{Rasch 0-10 scale}_{\mathbf{pain}}\mathbf{+item 5+}\mathbf{Rasch 0-10 scale}_{\mathbf{stiffness+}}\mathbf{Rasch 0-10 scale}_{\mathbf{impact of symptoms+}} \mathbf{Rasch 0-10 scale}_{\mathbf{psychological aspects}}}{\mathbf{5}}$

**FOACS_RASCH _hip OA_ =** $\frac{\mathbf{Rasch 0-10 scale}_{\mathbf{pain}}\mathbf{+}\mathbf{Rasch 0-10 scale}_{\mathbf{stiffness+}}\mathbf{Rasch 0-10 scale}_{\mathbf{impact of symptoms+}} \mathbf{Rasch 0-10 scale}_{\mathbf{psychological aspects}}}{\mathbf{4}}$
